# Selective estrogen receptor degraders and their effect on the breast cancer immune microenvironment

**DOI:** 10.1172/JCI207633

**Published:** 2026-10-15

**Authors:** Kristen S. Young, Sean W. Fanning

**Affiliations:** Department of Cancer Biology, Loyola University Chicago, Stritch School of Medicine, Maywood, Illinois, USA.

## Abstract

One in eight women will be diagnosed with breast cancer in their lifetime. Most breast cancers express estrogen receptor α (ERα) and depend on its signaling, which promotes tumor growth, survival, and metastatic spread through hormone-dependent transcriptional reprogramming. Selective estrogen receptor degraders/downregulators (SERDs), such as fulvestrant (faslodex), which was FDA approved in 2002, are second-line endocrine therapies for the treatment of advanced metastatic disease. Tumors containing activating hotspot mutations in *ESR1* (the gene encoding ERα) retain some sensitivity to fulvestrant but require higher doses to achieve inhibition of the mutant receptor. Substantial efforts to develop next-generation SERDs with improved pharmacology over fulvestrant have led to the FDA approval of the first-in-class orally available SERD elacestrant (Orserdu), followed by imlunestrant (Inluriyo), and the anticipated approval of several more therapies. Excitingly, recent studies show that in addition to their previously described antitumoral mechanisms, SERDs also act by increasing antitumor immunity. In this Review, we detail the current SERD clinical landscape and our emerging understanding of the interplay among SERDs, breast cancer cells, and the tumor immune microenvironment.

## Introduction

In the United States, breast cancer (BC) is the leading cause of cancer-related deaths among women under 50, with more than 40,000 deaths projected in 2026 (1). Unique molecular features classify BCs into three main subtypes: estrogen receptor α positive (ER^+^), human epidermal growth factor-2 positive (HER2^+^), and triple-negative BC (TNBC) (2). Most BCs (about 70%–80%) are ER^+^; this classification denotes that they overexpress the oncogenic nuclear transcription factor (3, 4). ER^+^ BCs are stimulated by the hormone estrogen; 17β-estradiol (E2) as the most potent endogenous estrogen; it elicits a robust upregulation of tumor-promoting genes (5). Endocrine therapy (ET) refers to a class of drugs that aim to slow tumor growth by blocking proproliferative E2-driven signaling. Here, we focus mainly on ET’s effect on the canonical ERα66, but it should be noted that other receptors are E2-responsive and may also be affected by ET. Another isoform, ERβ, also binds E2, and there are variants of ERα, including ERα46 and ERα36, whose distribution and function are detailed in a recent review (6). G protein–coupled estrogen receptor (GPER, also known as GPR30) also acts as an E2 agonist in syngeneic BC murine studies and binds anti-estrogens (7, 8).

First-line ERα-targeted ETs belong to two drug classes, which have distinct mechanisms of action. Aromatase inhibitors (AIs) starve ERα of its endogenous hormone by preventing the conversion of androgens into E2 (9, 10). AIs are the standard of care (SOC) in postmenopausal patients who have not received prior ET (11). In premenopausal patients, the first-line ET is the selective estrogen receptor modulator (SERM) tamoxifen, which is prescribed with or without ovarian suppression (12). The major active metabolite of tamoxifen is 4-hydroxytamoxifen (4OHT), which is a competitive allosteric ER antagonist in BC cells that acts as a weak estrogen in other tissues such as the uterus and bone (13, 14). These first-line ETs are administered for 5–10 years in the adjuvant setting to reduce the risk of recurrence (15–17).

Nearly half of all patients with BC with high nodal involvement at the time of primary diagnosis will relapse in their lifetime (18). This recurrent disease has often metastasized to distant organs at the time of diagnosis, most commonly to the bone, liver, lung, and brain (19, 20). Over 95% of BCs retain their ER^+^ or ER^–^ status in the metastatic setting, as measured by immunohistochemistry (21). However, approximately 40% of patients with metastatic ER^+^ BC present with hotspot somatic mutations to the gene encoding ERα, *ESR1*, with the most prevalent being a tyrosine-to-serine missense mutation at position 537 (Y537S) and a glutamate-to-glycine missense mutation at position 538 (D538G) (22–25). These mutations are highly enriched in metastatic lesions, but less than 1% of ET-naive patients have detectable *ESR1* mutations, suggesting that they arise over time from a dormant disease state (26). These mutations stabilize a constitutively active receptor, enabling ERα to stimulate gene transcription in the absence of E2 (27, 28).

Beyond promoting a constitutively active receptor, *ESR1* mutations increase the oncogenicity of ER^+^ BCs. Mutant ERα recruits transcriptional coactivator proteins to drive both canonical and pathogenic transcriptional programs (29). BC cells that harbor *ESR1* mutants display increased Histone H3 Lysine 27 acetylation (H3K27ac), corresponding to enhanced chromatin accessibility, which enables the expression of additional genes associated with metastatic aggressiveness (29). *ESR1* mutants upregulate genes associated with cell-cell adhesion and downregulate genes related to the extracellular matrix, which cumulatively enhances tumor extravasation, cell clumping in circulation, and invasion and survival in distant organs (30).

The most prevalent *ESR1* mutations, Y537S and D538G, also impact ET efficacy in BC cells. Both mutations decrease the ligand-binding affinity of ERα’s ligand-binding domain (LBD), requiring higher doses of ETs to achieve antiproliferative activities (27, 28, 31, 32). In the case of tamoxifen, maximal dosing does not fully suppress Y537S ERα transactivation within BC cells (27, 28). Reduced tamoxifen efficacy stems from mutational influences on the capacity of ETs to exert the allosteric structural rearrangements required to exert their antagonistic activities (27, 31, 33). Collectively, *ESR1* mutations confer ET resistance by engaging canonical and neomorphic biologic mechanisms of action, while also reducing the target engagement capacity of clinically approved first-line ETs.

Selective estrogen receptor degraders/downregulators (SERDs) were developed as second-line therapies for metastatic ER^+^ BC. Classically, SERDs induce the antagonistic conformation of ERα to oppose estrogenic gene expression programs before destabilizing the receptor, leading to ubiquitination and proteasomal degradation (31, 34–37). In advanced disease, the SERD fulvestrant acts as a complete antiestrogen throughout the body (38). While fulvestrant demonstrates superior efficacy compared with tamoxifen in models harboring *ESR1* mutations in vitro, its utility is limited by its poor solubility (39–41). Next-generation SERDs have been developed to target mutant ERα across all metastatic sites with improved pharmacologic properties, especially solubility and oral availability. The past few years have brought tremendous advances in the development of next-generation SERDs and, with them, an improved understanding of how to target ERα in complex tumor microenvironments. Here, we summarize the current clinical landscape of SERDs for the treatment of BC (Table 1) and highlight recent advances in our understanding of how they reshape the tumor microenvironment to enhance anticancer immunity.

## Current FDA-approved SERDs for ER^+^ BC

### Fulvestrant.

Fulvestrant was the first SERD to receive FDA approval and is a complete ERα antagonist throughout the body, administered via an intramuscular injection owing to its poor solubility and lack of oral bioavailability (39, 40). Fulvestrant has been shown to have the same efficacy as anastrozole (a common AI) in advanced ER^+^ BCs (42). Initially, AIs were used as second-line therapy after tamoxifen treatment for recurrent disease in postmenopausal patients with ER^+^ BC. However, in the early 2000s, third-generation AIs (anastrozole, letrozole, and exemestane) were approved for first-line ET, demonstrating improved disease-free survival and reduced recurrence compared with tamoxifen in this patient population (43). Tamoxifen remains the SOC ET in premenopausal patients owing to its ability to prevent recurrence, with a disease-free 8-year survival rate of 78.9%–83.2% (44). While the phase III FALCON Trial reported a progression-free survival (PFS) benefit in ET-naive postmenopausal women receiving fulvestrant compared with anastrozole, there was no significant difference in overall survival (OS) (45). These data support the use of fulvestrant as a second- or later-line ET if other lines fail.

Fulvestrant represents a major innovation in the use of ERα-targeted ETs across disease stages. Although response rates between fulvestrant and tamoxifen are similar in patients with previously untreated advanced ER^+^ disease, with objective response rates (ORRs) of 33.2% and 31.1%, respectively, fulvestrant shows increased efficacy in in vitro models and in patients with tamoxifen resistance (46–48). However, fulvestrant also has common life-impacting side effects, including injection site reactions, gastrointestinal adverse events, and joint pain (42, 49). In addition, BC cells harboring the most common *ESR1* mutations require elevated doses of fulvestrant to achieve complete transcriptional antagonism, exposing its poor solubility as a major liability in this setting (28, 32). Indeed, in metastatic clinical cohorts, patients with the Y537S *ESR1* mutation had a PFS of only 1.8 months on fulvestrant versus 3.5 months in patients with other detected *ESR1* mutations, and the median PFS with fulvestrant monotherapy, regardless of mutational status, is 3.18 months (50, 51). Responses are also nondurable, with a median OS of 26.4 months for the 500 mg dose reported in the CONFIRM trial (52). While fulvestrant remains an essential second-line ET, these studies showed the need for continued SERD development.

### Elacestrant.

Elacestrant (Orserdu) is the first orally available SERD to be FDA approved for ER^+^/HER2^–^ advanced BC (53). Elacestrant is a chemically unconventional SERD, as its side arm attaches to a distinct ring, allowing a unique interaction with helix 8 of ERα, whereas SERD side arms are usually attached to the steroidal ring (33). Elacestrant exposes a unique protein-binding surface when bound to ERα, affecting the repertoire of coregulators that drive ligand-specific gene expression programs, thus leading to a neomorphic transcriptional program (33, 54). Using a library of other unconventional SERD scaffolds, the Nettles and Katzenellenbogen laboratories showed that targeting unique combinations of structural features allows ERα to bind unique transcriptional coregulator proteins (55). In addition, our group showed that perturbing other ERα structural motifs allows it to engage new and therapeutically relevant transcriptional programs in hormone-sensitive and -resistant BC cells (33, 56). Accordingly, an x-ray cocrystal structure of elacestrant with the ERα LBD shows an unconventional ligand-binding pose that interacts with some structural features unique to the molecule (57), allowing it to maintain antiproliferative activities in models of CDK4/6 inhibitor–resistant (CDK4/6i-resistant) BCs (58).

The therapeutic potential of elacestrant was first described in mammary xenograft murine tumor studies in 2015 (54). These studies ultimately led to the EMERALD phase III clinical trial, in which elacestrant demonstrated a significant increase in 6-month PFS compared with SOC ET (34.3 versus 20.4 months, respectively) and specifically showed a benefit in patients who harbored a detectable *ESR1* mutation (40.8 versus 19.1 months, respectively) (59, 60). This clinical trial required patients to have prior exposure to a CDK4/6i and at least one chemotherapy (60). In *ESR1* D538G- and Y537S/N-mutated tumors that received prior ET and CDK4/6i for at least 12 months, the median PFS for elacestrant was 8.6 months versus 1.9 months for SOC (AI or fulvestrant) (61). Mechanistically, elacestrant has notable antitumoral effects in murine mammary xenograft and subcutaneous patient-derived xenograft (PDX) tumor models and downregulates ERα levels through receptor degradation; however, it does show weak endometrial stimulation in uterine wet weight studies, suggesting that it is not a complete ERα antagonist (54, 62). Maintenance of at least some tissue-specific ERα pharmacology may have bone-sparing effects (63). Furthermore, elacestrant crosses the blood-brain barrier (BBB) in healthy postmenopausal women (64). The ability of a systemic SERD to cross the BBB is promising in combating brain metastases, but more studies are needed to understand how these molecules affect ERs throughout this complex environment (65).

Together, these studies suggest that elacestrant can engage neomorphic anticancer transcriptional activities by forming new coregulator complexes, raising the exciting possibility of an expanded ERα pharmacology that could improve treatment options for specific, molecularly defined patient populations, including those with detectable *ESR1* mutations.

### Imlunestrant.

Imlunestrant (Inluriyo) recently became the second orally available SERD to be FDA approved. It is specifically indicated for patients with advanced ER^+^/HER2^–^ BC with a detectable *ESR1* mutation (66). Molecular docking shows imlunestrant binds with high affinity to the Y537S ERα LBD, but unlike other SERDs, there is no predicted binding to D351 of ERα, and it is predicted to perturb the loop preceding helix 12 rather than the helix itself (67). Imlunestrant harbors a steroidal core, similar to fulvestrant but antagonizes ERα more efficiently. Imlunestrant is a pure antagonist at all concentrations, causing ER degradation in BC cells (67).

In the EMBER-3 phase III study, imlunestrant was administered with or without a CDK4/6i in pre- and postmenopausal patients who had progressed after starting an AI and had ER^+^/HER2^–^ disease (68). As seen with elacestrant, the greatest benefit was in patients with detectable *ESR1* mutations, with a median PFS of 5.5 months compared with 3.8 months with SOC (68). Data on imlunestrant’s benefits in patients with specific *ESR1* mutants was not specified. Notably, the EMBER-3 phase III study did not demonstrate a benefit for imlunestrant over SOC in the overall patient population, with a median 6-month PFS of 5.6 months versus 5.5 months with SOC (68). Imlunestrant can cross the BBB and markedly increases survival in a preclinical model of immunocompromised mice with ER^+^ intracranial tumors (69). It does not show tissue-specific partial agonist activities in immunocompromised PDX models (67). To this end, the characterization of bone-sparing activities of imlunestrant thus far is limited.

## SERDs in clinical trials for late-stage disease

### Giredestrant.

Several more SERDs are nearing FDA approval. Among these, giredestrant is a nonsteroidal oral SERD currently in phase III clinical trials (lidERA trial, NCT04961996). Preclinical in vitro studies showed that giredestrant has a potent antiproliferative IC_50_ and improved binding affinity to ERα compared with fulvestrant (70). In murine uterine wet weight studies, giredestrant was confirmed as an ERα degrader in the endometrium (70, 71). Furthermore, giredestrant reduced ERα levels in mouse mammary tissue in transgenic murine models with inducible WT or mutant *ESR1*, whereas fulvestrant exhibited varying results (71). Additionally, in an in vitro functional luciferase assay that quantified the interaction between ERα and steroid receptor coactivator-3 (SRC-3), giredestrant showed superior potency toward WT ERα, while retaining activity in the presence of the *ESR1* mutant compared with other clinically relevant molecules (56).

Important clinical trials are also underway to test whether giredestrant, and therefore other next-generation SERDs, could be used at earlier disease stages. These trials are anticipated to be completed in 2026 (72). In the early-stage trials, giredestrant is being compared with the physician’s choice ET for 5 years in the adjuvant setting (72). The phase II clinical trial coopERA compared giredestrant versus AI (anastrozole) in ET-naive, postmenopausal women for 2 weeks followed by 16 weeks of the same therapy with addition of a CDK4/6i (73). The Ki67 (proliferative) index of the tumor was reduced in the giredestrant versus the anastrozole group, and both groups had an increased benefit with the addition of CDK4/6i, with comparable adverse events (73). In the phase II aceIERA clinical trial, giredestrant was compared with SOC (fulvestrant or AI) in patients with advanced ER^+^ BC who had progressed after 1–2 lines of therapy, including patients treated with fulvestrant (74). There was no significant increase in PFS from this clinical trial in patients receiving giredestrant versus SOC (74). Furthermore, there was no statistical benefit in patients with detectable *ESR1* mutations; however, it should be noted that this cohort was smaller than that of patients with WT *ESR1* (90 patients versus 142, respectively) (74). In the phase III persevERA study in patients with locally advanced or metastatic ER^+^/HER2^–^ BC, giredestrant with CDK4/6i was compared with AI with CDK4/6i as a first-line therapy, but primary statistical endpoints were not met (75). However, in the lidERA study, giredestrant in the adjuvant setting significantly improved patient outcomes compared with SOC (tamoxifen or AI), although the data are still immature (76). This shows that giredestrant has promise as the first SERD that could be used consistently in the first-line setting.

### Camizestrant.

Camizestrant is also under clinical investigation for advanced ER^+^/HER2^–^ BC with detectable *ESR1* mutations (77). In mechanistic studies, camizestrant can block ER transcriptional pathways in fulvestrant-insensitive immunocompromised PDX murine tumor models, showing retained ER antagonism (78). Structurally, camizestrant disrupts helix 12 stabilization like other SERDs; however, it retains activity in *ESR1-*mutant PDX murine models (78, 79). In the phase II SERENA-2 study, three doses of camizestrant were compared with fulvestrant in postmenopausal patients with metastatic or recurrent ER^+^/HER2^–^ BC who have progressed on no more than one ET in the advanced setting (80). A significant increase in median PFS was observed with the two lower doses of monotherapy (75 mg and 150 mg camizestrant) compared with fulvestrant (7.2 months, 7.7 months, and 3.7 months, respectively) in patients with WT and *ESR1* mutant BC, with superior efficacy over fulvestrant in patients with ESR1 mutations (median PFS, 6.3, 9.2, and 2.2 months, respectively) (80). In the phase III (SERENA-6) clinical trial, treatment with camizestrant is initiated upon detectable *ESR1* mutation (measured from circulating tumor DNA [ctDNA]) after receiving at least 6 months of AI and CDK4/6i, and the CDK4/6i is maintained during treatment (77). So far, median PFS has been significantly higher in the camizestrant arm than the SOC AI treatment arm (16.0 versus 9.2 months) (77).

In the CAMBRIA-1 and CAMBRIA-2 phase III clinical trials, camizestrant is compared with AI or tamoxifen therapy in patients with ER^+^/HER2^–^ early BC, as a first-line ET (81). CAMBRIA-1 compares 5 years of camizestrant versus standard ET for patients who have already received 2–5 years of standard adjuvant ET and have a high risk of recurrence, and prior CDK4/6i use is allowed (82). CAMBRIA-2 compares camizestrant as a first-line adjuvant ET versus standard ET for 7 years with CDK4/6i treatment for the first 2 years in both treatment arms (81). Preliminary findings for these studies will be completed in 2027.

### Vepdegestrant.

Vepdegestrant is a proteolysis-targeting chimera (PROTAC) rather than a traditional SERD and was recently FDA approved after its phase III trial, the VERITAC-2 study, in patients with advanced ER^+^/HER2^–^ BC harboring *ESR1* mutations that have received prior CDK4/6i and ET (83). Like SERDs, the greatest benefit of the PROTAC was seen in the PFS of the patient population harboring *ESR1* mutations (median PFS of 5.0 months versus 2.1 months with fulvestrant, *n* = 270), while no significant change was observed between the general population on vepdegestrant versus fulvestrant (3.8 months versus 3.6 months median PFS, respectively) (83).

PROTACs are heterobifunctional molecules that work by conjugating the protein of interest, in this case ERα, with a ubiquitin-ligase (E3) ligand, which directs ERα for degradation by the ubiquitin-proteasome system (84). Preclinical studies showed that vepdegestrant achieved a maximal degradation of ERα of greater than 90% in most WT *ESR1* ER^+^ cell lines (85). Further characterization showed a dose-dependent, antiproliferative effect with vepdegestrant in vitro in WT and mutant *ESR1* cell models, with more potent antiproliferative effects in the hormone-dependent WT models (85). In *ESR1*-mutant PDX murine models, treatment with vepdegestrant induced tumor regression, whereas fulvestrant only slowed tumor growth relative to vehicle (85). Furthermore, synergy was observed with the treatment of vepdegestrant in orthotopic xenograft murine models with the addition of CDK4/6i, alpelisib (PI3-kinase inhibitor), or evorlimus (mTOR inhibitor) (85). One consideration with vepdegestrant is liver metabolism, which may affect combination therapies due to drug-drug interactions (86). These preclinical and clinical trial results for the compound show that vepdegestrant is efficacious in advanced *ESR1-*mutant BC (similar to SERDs) (87).

### Palazestrant.

Palazestrant (OP-1250) is a SERD currently in phase III clinical trials in combination with a CDK4/6i as a first-line therapy for advanced ER^+^ BC (88). In the phase III study, which began recruitment in late 2025, palazestrant and CDK4/6i will be compared with an AI with a CDK4/6i (88). In the phase I/II study, the median PFS was 4.8 months overall and 5.6 months for patients with *ESR1* mutants with the 120 mg/d dose of palazestrant (89). Palazestrant is a complete estrogen receptor antagonist (CERAN), with brain penetrance and superior kinetics to fulvestrant in cell-line and PDX mammary and intracranial models (90). Furthermore, in vivo PDX studies show a greater benefit of palazestrant in combination with CDK4/6i (90). Palazestrant is characterized as a CERAN because of its ability to completely antagonize the AF-1 and AF-2 domains of ERα (34).

### Differential gene regulation and degradation patterns by SERDs.

Although all SERDs antagonize ERα, transcriptional responses arising from induced conformational changes to ERα are also critical for SERD efficacy. As such, in vitro studies show that, when fulvestrant binds to ERα, it induces conformational changes that blunt E2-driven transcription without requiring proteasomal degradation (37). Mechanistically, fulvestrant decreases the nuclear mobility of ERα, increasing ERα replacement (91). Unlike fulvestrant, which is a pure ERα degrader, elacestrant has partial agonistic effects on tumor growth in mammary PDX murine studies at low concentrations (54, 92). At higher concentrations in vitro, elacestrant downregulates the *ESR1* target genes *TFF1*, *PGR*, and *FHL1*, but at low concentrations in MCF7 mammary xenografts, it induces genes associated with tamoxifen resistance, such as *AGR2* and *KRT13* (54). In contrast, imlunestrant shows increased ERα degradation at low levels compared with elacestrant, and superior progesterone receptor (PR) inhibition compared with elacestrant in vitro (69). Interestingly, transcriptomic studies with Y537S *ESR1* PDX murine tumors show that imlunestrant more significantly downregulates estrogen response late genes, Myc targets, and cell cycle pathways and uniquely upregulates NF-κB and IFN-γ responses compared with fulvestrant (67). These data show that SERDs can engage distinct therapeutic mechanisms of action to achieve therapeutic efficacy in the *ESR1* mutant setting.

Genome-wide analyses further distinguish distinct SERD transcriptional effects. MCF7 cells treated with giredestrant show differential expression of over 2,400 genes, reduced accessibility of transcription start sites of E2 genes and H3K27ac, and increased expression of tumor suppressors, furthering the concept that ERα immobilization may be as important as degradation in antitumor control (93). Similarly, camizestrant specifically downregulates ER-early and -late gene signatures more robustly than fulvestrant, leading to utility in fulvestrant-resistant PDX murine models (78). The superior antagonism of ERα is the major contributor to camizestrant’s efficacy in the ESR1-mutant and fulvestrant-resistant settings (78).Thus, in addition to ERα antagonism, specific transcriptome changes are associated with the efficacy of SERDs. Together, these studies suggest that through engaging different therapeutic mechanisms of action, precision oncology approaches could improve efficacy by matching the patient with the SERD best suited to act in their unique molecular landscape. However, more work is needed to understand the SERD-specific ERα complexes that drive therapeutic response.

## SERDs and the tumor immune microenvironment

### The immune landscape of ER^+^ tumors.

Immune cells are critical mediators of both pathogenic and antitumor responses. Immunotherapies, which aim to reinvigorate antitumor immunity, have revolutionized care across multiple cancer types (94). These immunotherapies predominantly target programmed death-ligand 1 (PD-L1) or cytotoxic T-lymphocyte antigen-4 (CTLA-4), which suppress/exhaust immune cells (95, 96). Therapeutic anti–PD-L1 antibodies bind to the suppressive ligand on tumor cells and allow improved recognition of tumor cells by cytotoxic T cells (97, 98).

Immune checkpoint blockade (ICB) has reduced benefit in patients with ER^+^ BC compared with patients with TNBC, where anti–PD-L1 therapy has shown increased pathological complete response (pCR) and event-free survival with neoadjuvant chemotherapy (99–101). In the phase Ib KEYNOTE-028 trial, the ORR of PD-L1^+^ patients with ER^+^/HER2^–^ advanced BC treated with pembrolizumab (anti–PD-L1 therapy) was 12%, and no pCR effect was reported (102). However, in patients with TNBC in the KEYNOTE-012 study, the ORR was 18.5% (103). Furthermore, PD-L1 positivity in patients with TNBC is significantly higher than in patients with ER^+^ BC, at 58.6% versus 19.4%, respectively, illustrating the immune-cold landscape of ER^+^ tumors (102, 103).

T cell–directed therapies face many challenges in ER^+^ BC thanks to their low mutational rate, reduced neoantigen expression, and the immune-suppressive actions of ERα (104–107). Moreover, PD-L1 expression is downregulated by ERα in tumor cells (108, 109), whereas PD-L1 expression is higher in ER^–^ tumors (110, 111), and tumors with lower ER positivity (1%–50% positivity) show more robust responses to ICB (112). In transgenic murine *ESR1-*mutant tumors, immune pathway activation and cytokine expression are decreased, emphasizing the aggressive, immunosuppressive nature of these tumors (113). In contrast, in vitro characterization and clinical studies show increased immune pathway activation in *ESR1-*mutant tumors, revealing model-dependent differences and suggesting a potential therapeutic vulnerability (114, 115). Together, these studies suggest that increased *ESR1* expression correlates with reduced sensitivity to ICB, but distinct *ESR1-*mutant immune signatures represent a promising therapeutic opportunity.

### The role of estrogen signaling in the ER^+^ tumor immune microenvironment.

In the ER^+^ BC tumor immune microenvironment (TIME), E2 exerts direct and indirect immunosuppressive influences (Figure 1). A preclinical study with transgenic mice and ex vivo coculture showed that E2-stimulated ERα in the myeloid compartment prevented the proliferation of CD4^+^ and CD8^+^ T cells (116). Furthermore, E2 promotes proliferation of immunosuppressive Tregs (117, 118). Early studies investigating B cells suggest that high B cell activity in patients with ER^+^ BC correlates with a better prognosis, and genes upregulated by E2 signaling in B cells decrease their receptor signaling (119, 120). Furthermore, E2 promotes IgG and IgM production in healthy human PBMCs, and ERα activation with E2 triggers autoimmunity in B cells in transgenic murine models (121, 122). The role of E2 in autoimmunity is under investigation.

E2 signaling is also immunosuppressive in innate immune compartments. ERα is expressed in NK cells and monocytes, though some cite lower levels of ERα in CD8^+^ T cells and monocytes compared with CD4^+^ T cells (123). Myeloid-derived suppressor cells (MDSCs) are innate immune cells that inhibit lymphocyte anticancer function in the immune microenvironment (124). E2 signaling increases the abundance of MDSCs via activation/phosphorylation of STAT3 in BC patient–derived bone marrow samples (125). In a syngeneic murine melanoma model, E2 signaling through ERα in myeloid cells fosters an immunosuppressive TIME that confers resistance to ICB (116). Eosinophils, which are innate granulocytes that interact with T cells, decrease in abundance upon E2 treatment and correspond with decreased expression of CCL11, CCL24, and CCL5, and ERα depletion within eosinophils decreases tumor growth in syngeneic mammary tumors and transgenic murine models (126, 127).

NK cells are innate immune lymphocytes that are critical for host defense and anticancer activities (128–130). Engagement of NK cell receptors, including NKG2D, NKp46, and CD16, can trigger direct cell killing (131, 132). Upon recognition, NK cells secrete cytokines, notably perforin and granzyme B, to kill cancer cells (129, 133). In BC, lower amounts of cytotoxic immune activity, including lower infiltrating NK effector activity and gene signatures, are observed in larger tumors with nodal involvement (134). In ER^+^/HER2^–^ BCs, NK cells are underactive, and, while higher infiltration is correlated with improved OS, low functionality and infiltration is noted in ER^+^/PR^+^ subtypes (135–137). The role of estrogen has been under investigation in NK cell biology. In NK cells from the uterus of human first-trimester decidua, treatment with E2 increased migration and proangiogenic vascular remodeling, which was blocked by fulvestrant administration (138). Estrogen suppresses NK cell–mediated cytotoxicity in vitro, and, perhaps accordingly, NK cells have lower activation levels in patients with ER^+^ BC compared with TNBC (135, 139). Specifically, E2 suppresses IFN-γ and IL-2 expression in murine NK cells and human PBMCs (140, 141). IFN-γ is critical for a robust NK cell–mediated antitumor response (142, 143).

Neutrophils and macrophages are phagocytic innate immune cells that have both tumor-promoting and killing activities. In murine ER^+^ xenograft and syngeneic BC models, E2 increases the abundance of tumor-associated neutrophil recruitment, which is opposed by fulvestrant (144). The influx of neutrophils is caused by an E2-dependent secretion of TGFβ1, an immunosuppressive cytokine, from the cancer cells, which attracts integrin lymphocyte function-associated antigen-1–positive (LFA-1^+^) neutrophils to the TIME (144). In immunocompetent postpartum mammary involution murine models supplemented with E2, neutrophils show increased expression of COX-2 and protumoral cytokines, including CXCL1, CXCL2, and S100A8 (145). E2 also increases recruitment and activation of protumor macrophages through CCL2 and CCL5 signaling in syngeneic murine mammary models, and these macrophages may be associated with enhanced cancer cell dissemination in a zebrafish model (146). In BC clinical datasets, inflammatory M1 polarization is associated with more aggressive, immunogenic cancer (147), and in preclinical melanoma murine models, E2 treatment promotes immunosuppressive M2 macrophage polarization (116). However, ER^+^ BC cells can induce inflammatory responses when cocultured with monocytes in full media (148). This discordance shows the context-dependent nature of macrophage polarization, suggesting that a spectrum of inflammatory and suppressive macrophage programs may exist in the ER^+^ TIME, which may be delineated as further immune-inclusive ER^+^ models are developed.

### The effect of SERDs on the TIME.

SERDs reinvigorate antitumor immunity in adaptive and innate immune compartments. Since fulvestrant is the longstanding SOC, most studies of the BC TIME have investigated this drug. In an ER^+^ syngeneic fulvestrant-resistant murine model, the combination of fulvestrant with ICB showed greater tumor regression than the single-agent alone, indicating that SERDs can affect the TIME to improve ICB efficacy (149). In an ER^–^ murine BC model, SERDs can act on ERα in MDSCs, blocking their accumulation and permitting influx of cytotoxic T cells (125). Furthermore, in immunocompetent murine models of ER^–^ BC, ICB in combination with fulvestrant or the SERM lasofoxifene increased antitumor activity, further suggesting that targeting ERα in nontumor cells enhances immunotherapy efficacy (126). Moreover, emerging sequencing data from the SOLTI-ELIPSE trial (NCT04797728) investigating adjuvant elacestrant treatment in early BC found that elacestrant induced genes related to immune activation, including *CD8A*, *IGJ*, *CD4*, and *GZMB* (150). This, combined with the finding that imlunestrant increases IFN-γ response, shows that antagonizing ERα in the TIME with SERDs improves the immunogenicity of ER^+^ tumors to improve ICB efficacy (67, 125, 149).

In vitro, pretreatment of BC cells with tamoxifen or fulvestrant increases NK cell–mediated killing of both ER^+^ and ER^–^ cell lines, an effect involving increased GPR30 signaling in the tumor cells (151). Similar results have been shown in cocultures of NK cells and lung cancer cells (152). This suggests that modulation of ERα in NK cells improves their cancer-killing capacities, but further studies are needed to define the complexity of ERα signaling in NK cells (151, 152).

In syngeneic murine models of melanoma, M2 polarization can be reversed by fulvestrant treatment (116). In addition, fulvestrant decreases the number of neutrophils in mammary tissue and the invasive margin of ER^+^ tumors compared with tamoxifen in normal mice as well as syngeneic and xenograft BC murine models, corresponding with an improved antitumor response (153, 154). Reduced macrophage infiltration in tumors was also observed with fulvestrant treatment (153). This indicates that antagonizing ER in these cells increases antitumor immunity by decreasing the prevalence of immunosuppressive cells in ER^+^ TIME.

## Discussion

While SERDs have historically been used as second-line ERα-targeted ETs, current clinical trials with giredestrant and camizestrant are testing whether SERDs can be moved to earlier disease stages. Next-generation SERDs have shown improved efficacy in the *ESR1* mutant setting and are orally available, thereby overcoming persistent limitations of fulvestrant (60). In 2023, elacestrant marked a major milestone, becoming the first orally available SERD that improved PFS in patients with metastatic BC, while specifically showing utility in patients harboring an *ESR1* mutation (54, 60, 155). Although a marked breakthrough, 6-month PFS is only extended by 13.9 months in the overall population (60). This highlights the aggressive nature of metastatic ER^+^ BC and the need for therapies that antagonize tumor cells and reengage the immune system to fight the disease.

Excitingly, some SERDs are brain penetrant, such as imlunestrant and elacestrant (64, 69). The role of E2 in ER^+^ brain metastasis is an active area of investigation. While studies in immunocompromised mice with intracranial tumors show decreased tumor burden when treated with a brain-penetrant SERD, and cocultures with TNBC cells and astrocytes show that E2 promotes brain metastatic colonization, there is also evidence that agonists for ERβ may increase BBB integrity (69, 156, 157). Therefore, further understanding of the complex relationship between E2, ERα, ERβ, and immune cells in the brain niche is needed. It should be noted as well that the SERM lasofoxifene is currently in phase III clinical trials in combination with a CDK4/6i for advanced ER^+^ *ESR1-*mutant BC (158). This is a competing strategy to SERDs in advanced ER^+^ BC, because lasofoxifene reinforces the inactive conformation of the Y537S ERα, leading to inhibition of xenograft tumor growth and metastasis in murine models (159, 160).

The roles of distinct immune cell compartments in ER^+^ BC remain difficult to resolve, as there is a paucity of ER^+^ syngeneic models. Therefore, immune-oncology studies typically use ER^–^ murine models or coculture systems. Furthermore, many preclinical ER^+^ models primarily focus on mammary tumors rather than metastatic progression and tumor cell colonization. Recently, a study utilizing matched patient ER^+^ biopsies from metastatic sites revealed trends in tumor-associated macrophages after treatment with ET^+^CDK4/6i that correlate with improved survival (161). Studies such as this that utilize next-generation sequencing techniques, such as single-cell RNA sequencing, are critical to delineate the TIME in advanced ER^+^ disease. Further research into the impact of SERDs on different cell types will enhance our understanding of their utility in patients and reveal new targets to combat the disease, and the emergence of next-generation SERDs provides more options to combat ER^+^ BC.

### Future therapeutic implications, directions, and outstanding questions.

Emerging preclinical evidence suggests that antagonizing the immune cell ER with SERDs enhances the antitumor response. Excitingly, studies in murine models of metastatic TNBC and colorectal cancer show that combining ICB with fulvestrant increases tumor-infiltrating lymphocytes and decreases M2 macrophages in the TIME (125, 162). In a rat ER^+^ BC model, fulvestrant alone promoted tumor regression, and in combination with ICB it reduced the emergence of more tumors (163). This illustrates the potential of combining ER degradation in the TIME with ICB to limit cancer spread.

As noted above, most of the experimental models investigating the role of SERDs on the TIME utilize fulvestrant, leaving a sizeable gap in our understanding of how different SERDs impact the TIME and different immune populations. However, state-of-the-art technologies including single-cell and spatial transcriptomics and imaging mass cytometry promise to improve our understanding of SERD influences on the TIME. As clinically relevant ER^+^ models continue to develop, beneficial combinations of SERDs with immunotherapies that have been revolutionary for other cancers may be illuminated for better disease management. This remains an exciting future direction in personalized medicine for ER^+^ BC.

## Conflict of interest

The authors have declared that no conflict of interest exists.

## Funding support

This work is the result of NIH funding, in whole or in part, and is subject to the NIH Public Access Policy. Through acceptance of this federal funding, the NIH has been given a right to make the work publicly available in PubMed Central.

- R37CA279341 to SWF.

## Figures and Tables

**Figure 1:**
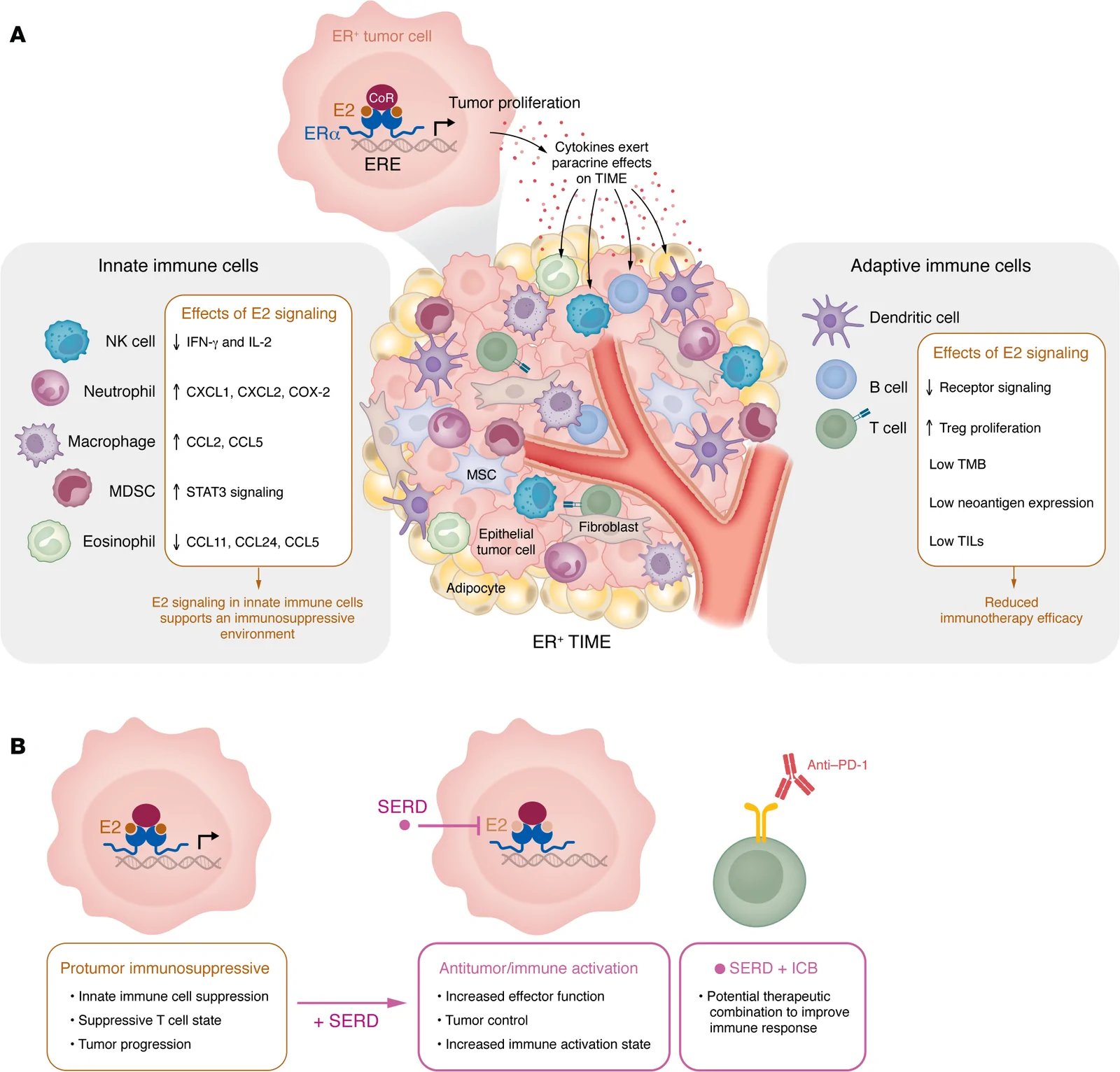
ERα signaling contributes to the immunosuppressive phenotype in ER^+^ breast cancer. ERα signaling contributes to the immunosuppressive phenotype in ER^+^ breast cancer. (**A**) In addition to the direct effects of E2 on tumor cell proliferation, the hormone also acts on the TIME. In innate immune cells (left), ERα signaling downregulates IFN-**γ** signaling and inflammatory chemokines. In the adaptive immune compartment (right), E2 signaling promotes Treg proliferation and decreases B cell receptor signaling. These effects contribute to the reduced efficacy of immunotherapy in ER^+^ BC. (**B**) SERDs can act on both tumor cells and the TIME. In addition to blocking the proliferative effect of E2 signaling in tumor cells, SERDs may make the TIME more immunogenic, increasing the efficacy of immunotherapy. ERE, estrogen response element; MSC, mesenchymal stem cell; TMB, tumor mutational burden; TIL, tumor-infiltrating lymphocyte.

**Table 1:**
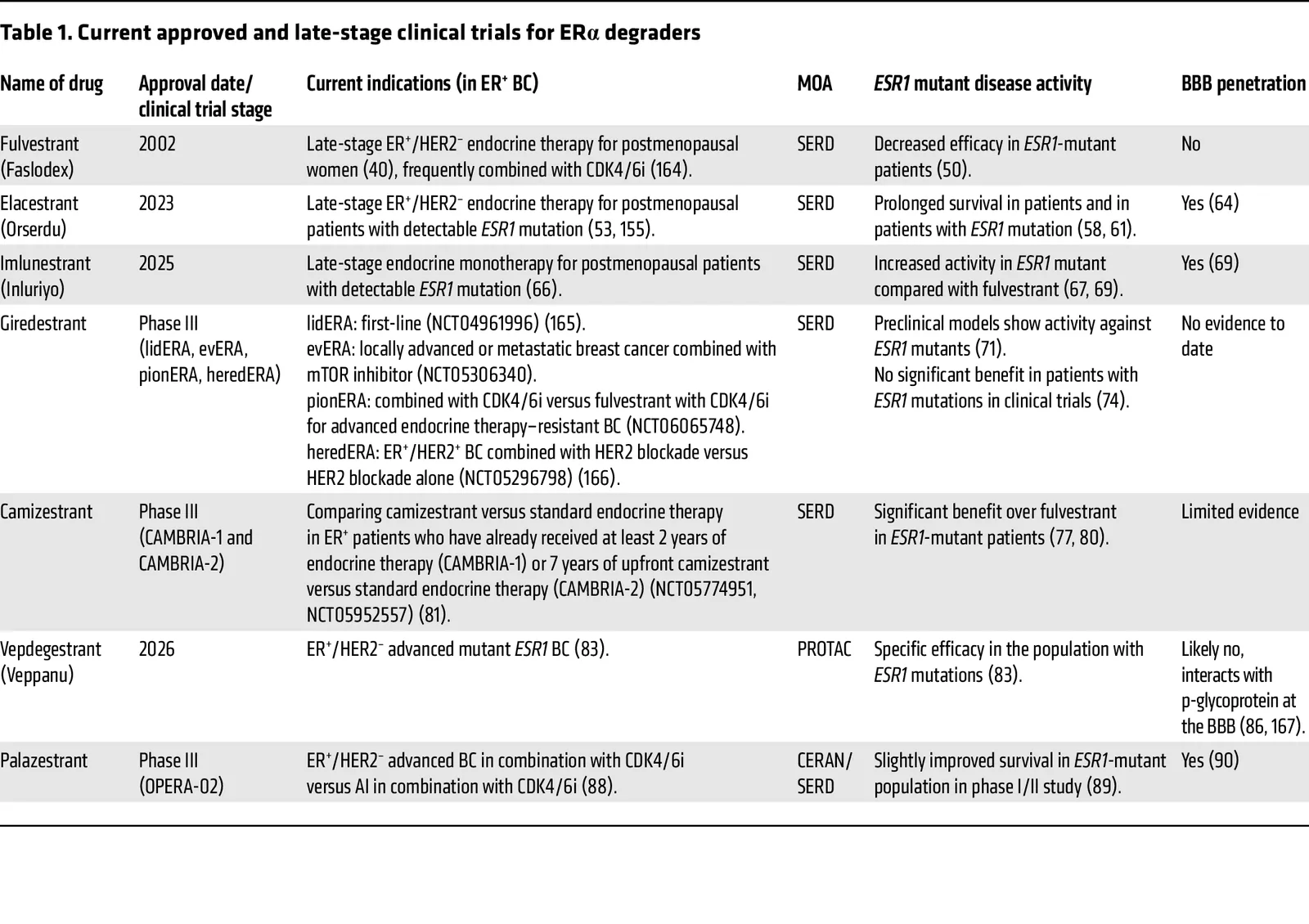
Current approved and late-stage clinical trials for ERα degraders

## Version 1 10/15/2026

Electronic publication

## References

[1] Siegel RL (2026). Cancer statistics, 2026. CA Cancer J Clin.

[2] Yersal O, Barutca S (2014). Biological subtypes of breast cancer:Prognostic and therapeutic implications. World J Clin Oncol.

[3] Jiang C (2024). Trends in breast cancer incidence by estrogen receptor status in the United States, 2004-2020. Int J Cancer.

[4] Li Y (2020). Clinicopathological characteristics and breast cancer-specific survival of patients with single hormone receptor-positive breast cancer. JAMA Netw Open.

[5] Frasor J (2003). Profiling of estrogen up- and down-regulated gene expression in human breast cancer cells: insights into gene networks and pathways underlying estrogenic control of proliferation and cell phenotype. Endocrinology.

[6] Chakraborty B (2023). Estrogen Receptor Signaling in the Immune System. Endocr Rev.

[7] Marjon NA (2014). G protein-coupled estrogen receptor regulates mammary tumorigenesis and metastasis. Mol Cancer Res.

[8] Thomas P (2005). Identity of an estrogen membrane receptor coupled to a G protein in human breast cancer cells. Endocrinology.

[9] Chumsri S (2011). Aromatase, aromatase inhibitors, and breast cancer. J Steroid Biochem Mol Biol.

[10] Ghosh D (2009). Structural basis for androgen specificity and oestrogen synthesis in human aromatase. Nature.

[11] Rugo HS (2016). Endocrine therapy for hormone receptor-positive metastatic breast cancer: American Society of Clinical Oncology Guideline. J Clin Oncol.

[12] Francis PA (2015). Adjuvant ovarian suppression in premenopausal breast cancer. N Engl J Med.

[13] Kübler K (2025). Tamoxifen induces PI3K activation in uterine cancer. Nat Genet.

[14] Turner RT (1987). Tamoxifen prevents the skeletal effects of ovarian hormone deficiency in rats. J Bone Miner Res.

[15] Davies C (2013). Long-term effects of continuing adjuvant tamoxifen to 10 years versus stopping at 5 years after diagnosis of oestrogen receptor-positive breast cancer: ATLAS, a randomised trial. Lancet.

[16] Burstein HJ (2014). Adjuvant endocrine therapy for women with hormone receptor-positive breast cancer: american society of clinical oncology clinical practice guideline focused update. J Clin Oncol.

[17] Goss PE (2016). Extending aromatase-inhibitor adjuvant therapy to 10 years. N Engl J Med.

[18] Pan H (2017). 20-year risks of breast-cancer recurrence after stopping endocrine therapy at 5 years. N Engl J Med.

[19] Zhang XH (2013). Metastasis dormancy in estrogen receptor-positive breast cancer. Clin Cancer Res.

[20] Wu Q (2017). Breast cancer subtypes predict the preferential site of distant metastases: a SEER based study. Oncotarget.

[21] Gomez-Fernandez C (2008). Immunohistochemically determined estrogen receptor phenotype remains stable in recurrent and metastatic breast cancer. Am J Clin Pathol.

[22] Jeselsohn R (2014). Emergence of constitutively active estrogen receptor-α mutations in pretreated advanced estrogen receptor-positive breast cancer. Clin Cancer Res.

[23] Chandarlapaty S (2016). Prevalence of ESR1 mutations in cell-free DNA and outcomes in metastatic breast cancer: a secondary analysis of the BOLERO-2 clinical trial. JAMA Oncol.

[24] Robinson DR (2013). Activating ESR1 mutations in hormone-resistant metastatic breast cancer. Nat Genet.

[25] Fribbens C (2016). Plasma ESR1 mutations and the treatment of estrogen receptor-positive advanced breast cancer. J Clin Oncol.

[26] Kinslow CJ (2022). Prevalence of estrogen receptor alpha (ESR1) somatic mutations in breast cancer. JNCI Cancer Spectr.

[27] Fanning SW (2016). Estrogen receptor alpha somatic mutations Y537S and D538G confer breast cancer endocrine resistance by stabilizing the activating function-2 binding conformation. Elife.

[28] Toy W (2013). ESR1 ligand-binding domain mutations in hormone-resistant breast cancer. Nat Genet.

[29] Jeselsohn R (2018). Allele-specific chromatin recruitment and therapeutic vulnerabilities of ESR1 activating mutations. Cancer Cell.

[30] Li Z (2022). Hotspot ESR1 mutations are multimodal and contextual modulators of breast cancer metastasis. Cancer Res.

[31] Fanning SW (2018). The SERM/SERD bazedoxifene disrupts ESR1 helix 12 to overcome acquired hormone resistance in breast cancer cells. Elife.

[32] Toy W (2017). Activating ESR1 mutations differentially affect the efficacy of ER antagonists. Cancer Discov.

[33] Hancock GR (2022). Unconventional isoquinoline-based SERMs elicit fulvestrant-like transcriptional programs in ER+ breast cancer cells. NPJ Breast Cancer.

[34] Ng RA (2025). Discovery of palazestrant (OP-1250), a potent and orally bioavailable complete estrogen receptor antagonist (CERAN) and selective estrogen receptor degrader (SERD). ACS Omega.

[35] Hosfield DJ (2022). Stereospecific lasofoxifene derivatives reveal the interplay between estrogen receptor alpha stability and antagonistic activity in ESR1 mutant breast cancer cells. Elife.

[36] Fanning SW (2018). Specific stereochemistry of OP-1074 disrupts estrogen receptor alpha helix 12 and confers pure antiestrogenic activity. Nat Commun.

[37] Wardell SE (2011). The turnover of estrogen receptor α by the selective estrogen receptor degrader (SERD) fulvestrant is a saturable process that is not required for antagonist efficacy. Biochem Pharmacol.

[38] Long X, Nephew KP (2006). Fulvestrant (ICI 182,780)-dependent interacting proteins mediate immobilization and degradation of estrogen receptor-alpha. J Biol Chem.

[39] Robertson JF, Harrison M (2004). Fulvestrant: pharmacokinetics and pharmacology. Br J Cancer.

[40] Bross PF (2002). FDA drug approval summaries: fulvestrant. Oncologist.

[41] Gelsomino L (2016). ESR1 mutations affect anti-proliferative responses to tamoxifen through enhanced cross-talk with IGF signaling. Breast Cancer Res Treat.

[42] Howell A (2002). Fulvestrant, formerly ICI 182,780, is as effective as anastrozole in postmenopausal women with advanced breast cancer progressing after prior endocrine treatment. J Clin Oncol.

[43] Mouridsen H (2001). Superior efficacy of letrozole versus tamoxifen as first-line therapy for postmenopausal women with advanced breast cancer: results of a phase III study of the International Letrozole Breast Cancer Group. J Clin Oncol.

[44] Francis PA (2018). Tailoring adjuvant endocrine therapy for premenopausal breast cancer. N Engl J Med.

[45] Robertson JFR (2025). Fulvestrant versus anastrozole in endocrine therapy-naïve women with hormone receptor-positive advanced breast cancer: final overall survival in the phase III FALCON Trial. J Clin Oncol.

[46] Michalides R (2004). Tamoxifen resistance by a conformational arrest of the estrogen receptor alpha after PKA activation in breast cancer. Cancer Cell.

[47] Howell A (2004). Comparison of fulvestrant versus tamoxifen for the treatment of advanced breast cancer in postmenopausal women previously untreated with endocrine therapy: a multinational, double-blind, randomized trial. J Clin Oncol.

[48] Howell A (1995). Response to a specific antioestrogen (ICI 182780) in tamoxifen-resistant breast cancer. Lancet.

[49] Sammons S (2019). Fulvestrant-based combination therapy for second-line treatment of hormone receptor-positive advanced breast cancer. Target Oncol.

[50] Kingston B (2024). ESR1 F404 mutations and acquired resistance to fulvestrant in ESR1-mutant breast cancer. Cancer Discov.

[51] Ogata N (2025). Efficiency of fulvestrant monotherapy after CDK4/6 inhibitor exposure: is this a viable choice?. Cancers (Basel).

[52] Di Leo A (2014). Final overall survival: fulvestrant 500 mg vs 250 mg in the randomized CONFIRM trial. J Natl Cancer Inst.

[53] Hoy SM (2023). Elacestrant: first approval. Drugs.

[54] Wardell SE (2015). Evaluation of the pharmacological activities of RAD1901, a selective estrogen receptor degrader. Endocr Relat Cancer.

[55] Min J (2021). Dual-mechanism estrogen receptor inhibitors. Proc Natl Acad Sci U S A.

[56] Young KS (2025). Targeting unique ligand binding domain structural features downregulates DKK1 in Y537S ESR1 mutant breast cancer cells. Breast Cancer Res.

[57] Fanning SW (2020). X-ray crystal structure analysis of elacestrant (RAD1901), a novel selective estrogen receptor degrader (SERD), bound to estrogen receptor alpha ligand binding domain. J Clin Oncol.

[58] Patel HK (2019). Elacestrant (RAD1901) exhibits anti-tumor activity in multiple ER+ breast cancer models resistant to CDK4/6 inhibitors. Breast Cancer Res.

[59] Bardia A (2019). EMERALD: Phase III trial of elacestrant (RAD1901) vs endocrine therapy for previously treated ER+ advanced breast cancer. Future Oncol.

[60] Bidard FC (2022). Elacestrant (oral selective estrogen receptor degrader) versus standard endocrine therapy for estrogen receptor-positive, human epidermal growth factor receptor 2-negative advanced breast cancer: results from the randomized phase III EMERALD Trial. J Clin Oncol.

[61] Bardia A (2024). Elacestrant in ER+, HER2- metastatic breast cancer with ESR1-mutated tumors: subgroup analyses from the phase III EMERALD trial by prior duration of endocrine therapy plus CDK4/6 inhibitor and in clinical subgroups. Clin Cancer Res.

[62] Bihani T (2017). Elacestrant (RAD1901), a selective estrogen receptor degrader (SERD), has antitumor activity in multiple ER(+) breast cancer patient-derived xenograft models. Clin Cancer Res.

[63] Börjesson AE (2011). Roles of transactivating functions 1 and 2 of estrogen receptor-alpha in bone. Proc Natl Acad Sci U S A.

[64] Conlan MG (2020). Pharmacokinetic and pharmacodynamic studies of elacestrant, a novel oral selective estrogen receptor degrader, in healthy post-menopausal women. Eur J Drug Metab Pharmacokinet.

[65] Wardell SE (2024). 8595 brain penetration impedes SERD efficacy in breast cancer pre-clinical models. J Endocr Soc.

[66] Keam SJ (2025). Imlunestrant: first approval. Drugs.

[67] Sherman S (2025). Imlunestrant a next-generation oral SERD overcomes ESR1 mutant resistance in estrogen receptor-positive breast cancer. JCI Insight.

[68] Jhaveri KL (2025). Imlunestrant with or without Abemaciclib in Advanced Breast Cancer. N Engl J Med.

[69] Bhagwat SV (2025). Imlunestrant is an oral, brain-penetrant selective estrogen receptor degrader with potent antitumor activity in ESR1 wild-type and mutant breast cancer. Cancer Res.

[70] Liang J (2021). GDC-9545 (Giredestrant): a potent and orally bioavailable selective estrogen receptor antagonist and degrader with an exceptional preclinical profile for ER+ breast cancer. J Med Chem.

[71] Liang J (2022). Giredestrant reverses progesterone hypersensitivity driven by estrogen receptor mutations in breast cancer. Sci Transl Med.

[72] https://clinicaltrials.gov/study/NCT04961996.

[73] Hurvitz SA (2023). Neoadjuvant palbociclib plus either giredestrant or anastrozole in oestrogen receptor-positive, HER2-negative, early breast cancer (coopERA Breast Cancer): an open-label, randomised, controlled, phase 2 study. Lancet Oncol.

[74] Martín M (2024). Giredestrant for estrogen receptor-positive, HER2-negative, previously treated advanced breast cancer: results from the randomized, phase II acelERA breast cancer study. J Clin Oncol.

[75] Turner NC (2026). Giredestrant (GIRE) + palbociclib (PALBO) vs letrozole (LET) + PALBO as first-line (1L) therapy in patients (pts) with estrogen receptor–positive, HER2-negative locally advanced or metastatic breast cancer (ER+, HER2– LA/mBC): Primary analysis of the phase III persevERA BC trial. J Clin Oncol.

[76] Bardia A (2026). Abstract GS1-10: Giredestrant vs standard-of-care endocrine therapy as adjuvant treatment for patients with estrogen receptor-positive, HER2-negative early breast cancer: Results from the global Phase III lidERA Breast Cancer trial. Clin Cancer Res.

[77] Bidard FC (2025). First-line camizestrant for emerging ESR1-mutated advanced breast cancer. N Engl J Med.

[78] Lawson M (2023). The next-generation oral selective estrogen receptor degrader camizestrant (AZD9833) suppresses ER+ breast cancer growth and overcomes endocrine and CDK4/6 inhibitor resistance. Cancer Res.

[79] Scott JS (2020). Discovery of AZD9833, a potent and orally bioavailable selective estrogen receptor degrader and antagonist. J Med Chem.

[80] Oliveira M (2024). Camizestrant, a next-generation oral SERD, versus fulvestrant in post-menopausal women with oestrogen receptor-positive, HER2-negative advanced breast cancer (SERENA-2): a multi-dose, open-label, randomised, phase 2 trial. Lancet Oncol.

[81] Hamilton EP (2025). CAMBRIA-1 & CAMBRIA-2 phase III trials: camizestrant versus standard endocrine therapy in ER+/HER2- early breast cancer. Future Oncol.

[82] https://clinicaltrials.gov/study/NCT05774951.

[83] Campone M (2025). Vepdegestrant, a PROTAC estrogen receptor degrader, in advanced breast cancer. N Engl J Med.

[84] Zhao L (2022). Targeted protein degradation: mechanisms, strategies and application. Signal Transduct Target Ther.

[85] Gough SM (2024). Oral estrogen receptor PROTAC vepdegestrant (ARV-471) is highly efficacious as monotherapy and in combination with CDK4/6 or PI3K/mTOR pathway inhibitors in preclinical ER+ breast cancer models. Clin Cancer Res.

[86] Yang DZ (2025). Effect of vepdegestrant, a PROTAC oestrogen receptor degrader, on dabigatran and rosuvastatin pharmacokinetics in healthy participants. Br J Clin Pharmacol.

[87] Ma Z, Zhou J (2025). NDA Submission of Vepdegestrant (ARV-471) to U.S. FDA: The Beginning of a New Era of PROTAC Degraders. J Med Chem.

[88] Tolaney SM (2026). OPERA-02: A phase 3 study of palazestrant plus ribociclib as first-line treatment of ER+, HER2- advanced breast cancer. J Clin Oncol.

[89] Hamilton EP (2025). Palazestrant, a novel oral Complete Estrogen Receptor Antagonist (CERAN) and Selective Estrogen Receptor Degrader (SERD), in patients with ER+/HER2- advanced or metastatic breast cancer: phase 1/2 study results. Breast Cancer Res.

[90] Parisian AD (2024). Palazestrant (OP-1250), a complete estrogen receptor antagonist, inhibits wild-type and mutant ER-positive breast cancer models as monotherapy and in combination. Mol Cancer Ther.

[91] Guan J (2019). Therapeutic ligands antagonize estrogen receptor function by impairing its mobility. Cell.

[92] Beumer JH, Foldi J (2023). Pharmacology and pharmacokinetics of elacestrant. Cancer Chemother Pharmacol.

[93] Zhou W (2026). Giredestrant immobilizes the estrogen receptor to exert potent antitumor efficacy against ER-active breast cancers. Cell Chem Biol.

[94] Wallis CJD (2019). Association of patient sex with efficacy of immune checkpoint inhibitors and overall survival in advanced cancers: a systematic review and meta-analysis. JAMA Oncol.

[95] Fallarino F (1998). B7-1 engagement of cytotoxic T lymphocyte antigen 4 inhibits T cell activation in the absence of CD28. J Exp Med.

[96] Latchman Y (2001). PD-L2 is a second ligand for PD-1 and inhibits T cell activation. Nat Immunol.

[97] Dong H (2002). Tumor-associated B7-H1 promotes T-cell apoptosis: a potential mechanism of immune evasion. Nat Med.

[98] Iwai Y (2002). Involvement of PD-L1 on tumor cells in the escape from host immune system and tumor immunotherapy by PD-L1 blockade. Proc Natl Acad Sci U S A.

[99] Xin Y (2021). Immune checkpoint inhibitors plus neoadjuvant chemotherapy in early triple-negative breast cancer: a systematic review and meta-analysis. BMC Cancer.

[100] Schmid P (2020). Pembrolizumab for early triple-negative breast cancer. N Engl J Med.

[101] Huo X (2021). Addition of immunotherapy to chemotherapy for metastatic triple-negative breast cancer: A systematic review and meta-analysis of randomized clinical trials. Crit Rev Oncol Hematol.

[102] Rugo HS (2018). Safety and antitumor activity of pembrolizumab in patients with estrogen receptor-positive/human epidermal growth factor receptor 2-negative advanced breast cancer. Clin Cancer Res.

[103] Nanda R (2016). Pembrolizumab in Patients With Advanced Triple-Negative Breast Cancer: Phase Ib KEYNOTE-012 Study. J Clin Oncol.

[104] Arima J (2026). ESR1 expression negatively correlates with immune cell infiltration and response to immune checkpoint inhibitors in estrogen receptor-positive/HER2-negative breast cancer. Ann Surg Oncol.

[105] Barroso-Sousa R (2020). Prevalence and mutational determinants of high tumor mutation burden in breast cancer. Ann Oncol.

[106] Palomeque J (2025). Estrogen receptor signaling drives immune evasion and immunotherapy resistance in HR+ breast cancer. J Clin Invest.

[107] Narang P (2019). The neoepitope landscape of breast cancer: implications for immunotherapy. BMC Cancer.

[108] Shuai C (2020). Estrogen receptor downregulates expression of PD-1/PD-L1 and infiltration of CD8(+) T cells by inhibiting IL-17 signaling transduction in breast cancer. Front Oncol.

[109] Hanamura T (2023). Expression of hormone receptors is associated with specific immunological profiles of the breast cancer microenvironment. Breast Cancer Res.

[110] Guo Y (2016). Prognostic and clinicopathological value of programmed death ligand-1 in breast cancer: A Meta-Analysis. PLoS One.

[111] Wimberly H (2015). PD-L1 Expression correlates with tumor-infiltrating lymphocytes and response to neoadjuvant chemotherapy in breast cancer. Cancer Immunol Res.

[112] Voorwerk L (2023). Immune landscape of breast tumors with low and intermediate estrogen receptor expression. NPJ Breast Cancer.

[113] Gu Y (2026). Activating mutations in ESR1 contribute to an immunosuppressive breast tumor microenvironment by dampening cytokine secretion. JCI Insight.

[114] De Marchi T (2024). Proteomic profiling reveals that ESR1 mutations enhance cyclin-dependent kinase signaling. Sci Rep.

[115] Li Z (2022). ESR1 mutant breast cancers show elevated basal cytokeratins and immune activation. Nat Commun.

[116] Chakraborty B (2021). Inhibition of estrogen signaling in myeloid cells increases tumor immunity in melanoma. J Clin Invest.

[117] Prieto GA, Rosenstein Y (2006). Oestradiol potentiates the suppressive function of human CD4 CD25 regulatory T cells by promoting their proliferation. Immunology.

[118] Tai P (2008). Induction of regulatory T cells by physiological level estrogen. J Cell Physiol.

[119] Lee S (2024). B-cell-mediated immunity predicts survival of patients with estrogen receptor-positive breast cancer. JCO Precis Oncol.

[120] Grimaldi CM (2002). Estrogen alters thresholds for B cell apoptosis and activation. J Clin Invest.

[121] Kanda N, Tamaki K (1999). Estrogen enhances immunoglobulin production by human PBMCs. J Allergy Clin Immunol.

[122] Hill L (2011). Differential roles of estrogen receptors α and β in control of B-cell maturation and selection. Mol Med.

[123] Phiel KL (2005). Differential estrogen receptor gene expression in human peripheral blood mononuclear cell populations. Immunol Lett.

[124] Srivastava MK (2010). Myeloid-derived suppressor cells inhibit T-cell activation by depleting cystine and cysteine. Cancer Res.

[125] Márquez-Garbán DC (2019). Antiestrogens in combination with immune checkpoint inhibitors in breast cancer immunotherapy. J Steroid Biochem Mol Biol.

[126] Artham S (2024). Estrogen signaling suppresses tumor-associated tissue eosinophilia to promote breast tumor growth. Sci Adv.

[127] Zhou J (2024). Eosinophils promote CD8(+) T cell memory generation to potentiate anti-bacterial immunity. Signal Transduct Target Ther.

[128] Moretta L (2002). Human NK cells and their receptors. Microbes Infect.

[129] Topham NJ, Hewitt EW (2009). Natural killer cell cytotoxicity: how do they pull the trigger?. Immunology.

[130] Anfossi N (2006). Human NK cell education by inhibitory receptors for MHC class I. Immunity.

[131] McGilvray RW (2009). NKG2D ligand expression in human colorectal cancer reveals associations with prognosis and evidence for immunoediting. Clin Cancer Res.

[132] Della Chiesa M (2006). The tryptophan catabolite L-kynurenine inhibits the surface expression of NKp46- and NKG2D-activating receptors and regulates NK-cell function. Blood.

[133] Byemerwa J (2025). The roles of natural killer cells in breast cancer pathobiology and their regulation by estrogens. Endocr Rev.

[134] Rezaeifard S (2021). Tumor infiltrating NK cell (TINK) subsets and functional molecules in patients with breast cancer. Mol Immunol.

[135] Liu Y (2024). Overexpressing S100A9 ameliorates NK cell dysfunction in estrogen receptor-positive breast cancer. Cancer Immunol Immunother.

[136] Nersesian S (2021). NK cell infiltration is associated with improved overall survival in solid cancers: A systematic review and meta-analysis. Transl Oncol.

[137] Bouzidi L (2021). Prognostic value of natural killer cells besides tumor-infiltrating lymphocytes in breast cancer tissues. Clin Breast Cancer.

[138] Gibson DA (2015). Estrogen-dependent regulation of human uterine natural killer cells promotes vascular remodelling via secretion of CCL2. Hum Reprod.

[139] Jiang X (2007). Interplay between the levels of estrogen and estrogen receptor controls the level of the granzyme inhibitor, proteinase inhibitor 9 and susceptibility to immune surveillance by natural killer cells. Oncogene.

[140] Hao S (2008). 17beta-estradiol suppresses cytotoxicity and proliferative capacity of murine splenic NK1.1+ cells. Cell Mol Immunol.

[141] Stopińska-Głuszak U (2006). Effect of estrogen/progesterone hormone replacement therapy on natural killer cell cytotoxicity and immunoregulatory cytokine release by peripheral blood mononuclear cells of postmenopausal women. J Reprod Immunol.

[142] Yi JI (2024). Interferon-gamma secretion is significantly decreased in stage III breast cancer patients. Int J Mol Sci.

[143] Zanker DJ (2021). Loss of type I IFN responsiveness impairs natural killer cell antitumor activity in breast cancer. Cancer Immunol Immunother.

[144] Vazquez Rodriguez G (2017). Estradiol promotes breast cancer cell migration via recruitment and activation of neutrophils. Cancer Immunol Res.

[145] Chung HH (2017). Estrogen reprograms the activity of neutrophils to foster protumoral microenvironment during mammary involution. Sci Rep.

[146] Svensson S (2015). CCL2 and CCL5 are novel therapeutic targets for estrogen-dependent breast cancer. Clin Cancer Res.

[147] Oshi M (2020). M1 Macrophage and M1/M2 ratio defined by transcriptomic signatures resemble only part of their conventional clinical characteristics in breast cancer. Sci Rep.

[148] Hollmén M (2015). Characterization of macrophage--cancer cell crosstalk in estrogen receptor positive and triple-negative breast cancer. Sci Rep.

[149] O’Leary KA (2023). Estrogen receptor blockade and radiation therapy cooperate to enhance the response of immunologically cold ER+ breast cancer to immunotherapy. Breast Cancer Res.

[150] Vidal M (2025). Elacestrant in women with estrogen receptor-positive and HER2-negative early breast cancer: results from the preoperative window-of-opportunity ELIPSE Trial. Clin Cancer Res.

[151] Wolfson B (2021). Exploiting off-target effects of estrogen deprivation to sensitize estrogen receptor negative breast cancer to immune killing. J Immunother Cancer.

[152] Hamilton DH (2016). Targeting estrogen receptor signaling with fulvestrant enhances immune and chemotherapy-mediated cytotoxicity of human lung cancer. Clin Cancer Res.

[153] Abrahamsson A (2020). Fulvestrant-mediated attenuation of the innate immune response decreases ER(+) breast cancer growth in vivo more effectively than tamoxifen. Cancer Res.

[154] Tower H (2022). Estrogen-induced immune changes within the normal mammary gland. Sci Rep.

[155] Shah M (2024). US Food and Drug Administration approval summary: elacestrant for estrogen receptor-positive, human epidermal growth factor receptor 2-Negative, ESR1-mutated advanced or metastatic breast cancer. J Clin Oncol.

[156] Sartorius CA (2016). Estrogen promotes the brain metastatic colonization of triple negative breast cancer cells via an astrocyte-mediated paracrine mechanism. Oncogene.

[157] Kirchner J (2024). Protecting the brain: novel strategies for preventing breast cancer brain metastases through selective estrogen receptor β agonists and in vitro blood-brain barrier models. Int J Mol Sci.

[158] Goetz MP (2025). ELAINE 3: phase 3 study of lasofoxifene plus abemaciclib to treat ER+/HER2-, ESR1-mutated, metastatic breast cancer. Future Oncol.

[159] Lainé M (2021). Lasofoxifene as a potential treatment for therapy-resistant ER-positive metastatic breast cancer. Breast Cancer Res.

[160] Hosfield DJ (2022). Stereospecific lasofoxifene derivatives reveal the interplay between estrogen receptor alpha stability and antagonistic activity in ESR1 mutant breast cancer cells. Elife.

[161] Strawser CH (2026). Single-cell transcriptomics reveals immune landscape dynamics in metastatic hormone receptor-positive breast cancer treated with abemaciclib and endocrine therapy. Clin Cancer Res.

[162] Benslimane Y (2024). Estrogen receptor blockade potentiates immunotherapy for liver metastases by altering the liver immunosuppressive microenvironment. Cancer Res Commun.

[163] Rojas-Jimenez E (2026). Evaluation of targeted and immune combination therapies in a rat model of hormone receptor-positive breast cancer. Proc Natl Acad Sci U S A.

[164] Turner NC (2018). Overall survival with palbociclib and fulvestrant in advanced breast cancer. N Engl J Med.

[165] https://www.aacr.org/about-the-aacr/newsroom/news-releases/novel-endocrine-therapy-giredestrant-improves-disease-free-survival-over-standard-of-care-for-patients-with-early-stage-breast-cancer-in-phase-iii-lidera-trial/.

[166] Kuemmel S (2024). heredERA Breast Cancer: a phase III, randomized, open-label study evaluating the efficacy and safety of giredestrant plus the fixed-dose combination of pertuzumab and trastuzumab for subcutaneous injection in patients with previously untreated HER2-positive, estrogen receptor-positive locally advanced or metastatic breast cancer. BMC Cancer.

[167] Chai AB (2022). Regulation of P-Glycoprotein in the Brain. Int J Mol Sci.

